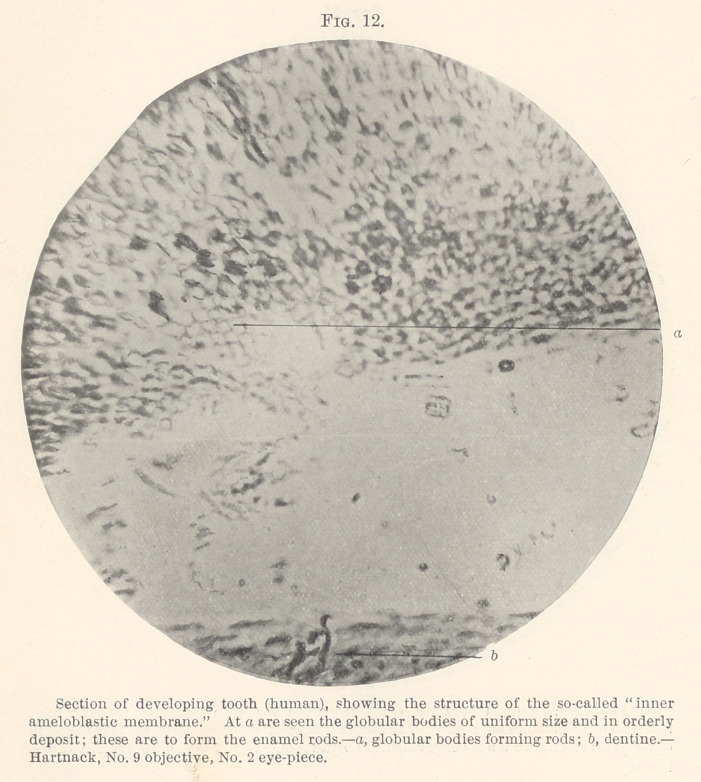# A Contribution to the Study of the Development of Dental Enamel

**Published:** 1897-04

**Authors:** R. R. Andrews


### International Dental Journal.

Vol. XVIII. April, 1897. No. 4.

### Original Communications.[1]

### A CONTRIBUTION TO THE STUDY OF THE DEVELOPMENT OF DENTAL ENAMEL.[2]

BY R. R. ANDREWS, A.M., D.D.S., F.R.M.S.

The subject which I have the honor to present to you this evening is on the finer processes taking place in the formation of enamel. A renewed interest in this subject has been shown since the recent publication of a series of papers by Dr. J. Leon Williams, of London. These papers are notable largely on account of their beautiful illustrations.[3] There are many photomicrographs of developing enamel taken with the finest modern high-power objectives, and photographed with a skill I have seldom seen surpassed. The story they tell is one that is very familiar to me, and I can endorse every point in the development of the enamel that they show. With my present knowledge I cannot coincide wholly with the subject-matter of the text. Some of the theories advanced by this author are new to the dental histologist.

In an address delivered at Atlanta, Georgia, in May last, before the American Medical Association, I criticised some of these con-

[1] The editor and publishers are not responsible for the views of authors of papers published in this department, nor for any claim to novelty, or otherwise, that may be made by them. No papers will be received for this department that have appeared in any other journal published in the country.

[2] Read before the New York Institute of Stomatology, January 5, 1897.

[3] The original negatives of the illustrations of this paper were broken during transportation some time ago. These have been copied from lantern slides, and do not show the structure as clearly as the originals.—R. R. A.

clusions, they representing, as I believe, only partial truths. If the author had been as cautious in the preparation of his series of papers as he afterwards was when answering an imperfectly printed abstract of my paper, containing type-writers' and proof-readers' blunders, the probabilities are there would have been less occasion for criticism. That there are mistakes and misinterpretations in his text is plainly evident to any one who understands the subject; but there is enough of value in this series of papers to make the whole dental profession his debtor, for the large amount of work he has performed and for the time he has devoted to the interests of our education. When considering the appearances of the tissue while studying enamel development it seems important that we should bear in mind the fact that we are necessarily compelled to use post-mortem tissue. We should work as near life as possible, preparing the tissue while it is yet warm from the mother, never allowing it to become dry, cutting our sections under fluid, and mounting them for study with very little further preparation. In studying these finer processes with the use of the higher powers of the microscope it is seldom necessary to stain the sections. They show their structure beautifully without stain. It will be a revelation to those working by the older methods to study tissues prepared in this simple way.

We will now ask your attention to the origin of the blood-supply to the enamel organ. We have been given to understand that there is an intricate plexus of blood-vessels developed in the enamel organ proper. Let us clearly understand this. The term "the enamel organ proper" describes it when the organ is in its perfected state, before calcification commences. It is at this time a kind of storehouse, having within the meshes of its so-called stellate reticulum enough calcific material to form the first layer of enamel. Its contents are wholly epithelial. I question how a plexus of blood-vessels—a connective-tissue structure—can be developed in this epithelial mass. Wedl, Magitot, Legros, and Sudduth have all failed to detect any within the enamel organ proper. Just at this time there are indications of folds of tissue like papilla and a forming plexus of blood-vessels in the connective tissue of the jaw over and entirely outside the enamel organ. These folds, I have supposed, were to be taken up by the expansion of the part by growth. Just outside of these folds, or papillæ, is seen a forming plexus of blood-vessels which is eventually to give the blood-supply to the enamel-forming layers. This is all outside of the enamel organ proper, and there exists between it and the stratum intermedium of the outer surface the cells of the external epithelium of the enamel organ. This layer afterwards disappears, and as calcification advances the plexus of blood-vessels is found to be against, and sometimes within, the stratum intermedium, which, with the ameloblasts, now become the enamel-forming layers. A plexus of blood-vessels has never been seen in the internal portion of the enamel organ proper.

Tomes's edition of 1876 says the outer surface of the enamel organ is indented by numerous papillary projections, into which enter blood-vessels; and Lionel Beale stated, thirty years ago, that a vascular net-work lies in the stratum intermedium. This was also seen and described in the developing tooth of the rat by Professor Howes and Mr. Poulton, two English observers, some years ago; but this, I believe, was after the enamel organ had disappeared from over the calcifying tooth-point.

The origin of the blood-supply is from the connective tissue. Salts of lime are given out or selected from the blood. Enamel, an epithelial structure, thus has its lime-supply from a connective-tissue source. The statement has been made that the blood-vessels are often seen to lie very near the ameloblasts, but never are found in actual contact. Dr. Sudduth states that it is *absolutely essential* that the capillary vessels should come in contact with the enamel cells before the process of calcification can be completed. I do not think that Dr. Sudduth meant the cells of the stratum intermedium, for he gave them another function, that of supplying new cells to the ameloblasts as the circumference of the enamel increases.

I shall next call your attention to the tissue Dr. Williams proposes to name the outer ameloblastic membrane. With my present knowledge I cannot consider this layer a membrane. In his reply to a criticism of mine in my address at Atlanta (November *Dental Cosmos*), Dr. Williams states, "I thought I made it clear in my paper that I was aware this appearance had not only been long known, but that it had also long been the subject of dispute and speculation." Does he make it clear? What did he say in his paper about this layer? (*Dental Cosmos* for February, 1896, p. 108.) "Thus we see we have a clear, sharply-marked, and differentiated line separating these two layers of cells (ameloblasts and stratum intermedium), which have *heretofore always been represented as closely connected.*"

I have recently investigated this "sharply-marked and differentiated line." It is not always constant. Where it does exist, it has the appearance of being a collection of fibres, and within the meshes of these fibres we find an accumulation of protoplasm, condensed, perhaps, by a post-mortem change, and this gives the appearance of a layer.

It is a disputed point whether fibres take their rise from a direct differentiation of the protoplasm, or whether this protoplasm is not converted into a homogeneous matrix from which the fibres differentiate. There can be no doubt but that the fibres are formed by a metamorphosis of the protoplasm, but I do not think they form within the cells. These fibres are formed, probably, from epithelial protoplasm. At various points they are connected with the ends of the cells of the stratum intermedium, on the one hand, and run into and between the ameloblasts on the other; and this was the appearance that gave me the impression that they had their origin from the stratum intermedium. Sometimes it is difficult to find any appearance of a layer; we see nothing but fibres; again it is quite marked, and in this condition it perhaps resembles a membrane. It is possible that the epithelial protoplasmic fibres which I described in my paper of 1890 have their origin here.

In regard to that structure called "the inner ameloblastic membrane," a structure between the ameloblasts and the formed enamel, he says, "It is impossible at present to speak definitely with regard to its origin, exact structure, or function. . . . It is possible that it plays an important part in the elaboration of material for enamel-building." This I am inclined to think is a mistake. The layer is an elaborated structure, a formed material consisting of a densified epithelial protoplasm; the substance that is to form the inter-enamel rod cement, and the calcific globular structures that are to form the enamel rods, together with the epithelial protoplasmic fibres which form the scaffolding that supports them. It is to all intents and purposes a formed material which further calcification will solidify into calcified enamel. As Dr. Sudduth has said, "Living matter cannot enter into chemical combination with inorganic matter as such, except the living lose its living principle and become non-living, formed material.

The layer is found to vary in thickness, sometimes being quite narrow, sometimes as thick as the layer of ameloblasts. These appearances show the various stages of the growth in the layer at the time when the section is cut. It is constant throughout the entire period of enamel growth. It seems impossible for us to understand why this fact should give a decided negative to the theory that ameloblasts are renewed from the stratum intermedium, as stated in the *Dental Cosmos* for February, page 111. It is probable that the allusion was meant for the so-called outer ameloblastic membrane, while describing the so-called inner one. I am led to believe that the appearance of membranes in both these layers is due to the fact that the protoplasm has condensed and jellied into

FIG. 1.

Section of tooth of calf, showing ameloblasts, also showing the minute globular bodies, calco-spherites, within the cells. There is no appearance of inner or outer membranes.—*a*, connective tissue of the jaw; *b*, ameloblasts containing calco-spherites; *c*, globular bodies, enamel globules.—Zeiss, 2 mm. objective, No. 12 eye-piece.

a line by reason of a post-mortem change within the meshes of the fibrous structure of each, causing the appearance of a discreet substance which shows a definite chemical reaction to the presence of certain stains. This inner layer is the so-called membrana præformativa of the earlier histologists. It is a partially calcified matrix material, somewhat resembling the substance we find everywhere on the border-land of calcification. It is seen occasionally in the first layers formed to double up on itself in folds, perhaps an excess of substance formed, to be smoothed out in the expansion by the growth of the dentine germ, when it will cover it as a single layer. I shall show this in my lantern exhibit.

Fig. 2.

Section of forming enamel (calf), showing the transition from globules into enamel rods. High power.—*a*, protoplasmic exudate, cement substance between the globules; *b*, the globular masses; *c*, the forming rods of enamel.—Zeiss, 2 mm. objective, No. 12 eye-piece.

In these papers there is given to the stratum intermedium as important a function, perhaps, as that which belongs to the ameloblasts. The stratum intermedium is a layer of cells between the ameloblasts and the stellate reticulum in the enamel organ proper, and later between the ameloblasts and the connective tissue of the jaw of the enamel-forming layers. In the early stages of enamel-formation I have considered this layer one of much importance, but I have not considered it as important a factor in the formation of the enamel as the ameloblasts. There can be no doubt that lime-salts from the blood are selected by these cells, but there is no optical evidence of any important chemical change towards forming calcific globules until it is absorbed in the protoplasm of the ameloblasts, which is really the enamel-builder. Quite recent investigation has led me to believe that protoplasm from the cells of the stratum intermedium may be deposited between this layer and the ameloblasts, and that this protoplasm becomes a tissue from which epithelial fibres are differentiated.

The ameloblasts, by a peculiar metamorphosis, have become specialized epithelial cells. In this condition they are unlike many other cells, being unable to lead an independent existence. They

Fig. 4.

Section of developing enamel (calf), showing globular bodies in the inter-rod cement substance, the large mass was caused by the coalescing of smaller ones, from the pressure on the cover glass of the specimen after mounting.—*a*, large globular mass in cement substance; *b*, forming layer of enamel; *c*, enamel formed.—Zeiss, 2 mm. objective, No. 12 eye-piece.

are cells without membranes at either end, and have rightly been called the modellers or builders of the enamel. The power of the protoplasm of these cells, with its formative activity, creates with the lime-salts of the blood bodies called calco-spherites. These are plainly seen to differ from the protoplasm, and by it they are placed so that they occupy a definite position, having a fixed form and structure. It will thus be seen that the ameloblasts absorb the calcific material from the blood; perhaps this is done by the cells of the stratum intermedium, and the ameloblasts absorb it from them. At any rate, within the ameloblast it is elaborated and given up to the calcifying surface in an altered form. A calcareous matrix is not formed out of protoplasm alone, but the protoplasm plays the part of an intermediary, selecting the substance from its environment. There are no reasonable grounds for the hypothesis that the phenomena of nuclear segmentation (mitosis of karyoki-

Fig. 5.

Section of forming enamel (calf), showing the transition of the globular bodies deposited by the ameloblasts into the rods of the enamel.—*a*, ameloblasts, torn away; *b*, globular bodies; *c*, the forming rods of enamel.—Hartnack, No. 9 objective, No. 2 eye-piece.

nesis) has anything to do with the forming of the lifeless, calcific substance which is to form the rods of the enamel. The phenomena which occur during nuclear segmentation are very complicated. Briefly, we may say in describing them that during the first stage the nucleus undergoes changes preparatory to division, resulting in the formation of nuclear segments and the nuclear centrosomes. At the same time the spindle commences to develop. During the second stage the nuclear segments, after the nuclear membrane has become dissolved, arrange themselves into a regular figure midway between the two poles, at the equator of the spindle. During the third stage the daughter segments, into which during one of the former stages the mother segments have divided by longitudinal fission, separate into two groups, which travel in opposite directions from the equator until they reach the neighborhood of the

FIG. 6.

Section of developing tooth (calf), showing the forming layer of enamel as wide as the layer of ameloblasts.—*a*, connective tissue; *b*, ameloblasts; *c*, forming enamel; *d*, dentine.—Hartnack, No. 9 objective, No. 2 eye-piece.

centrosomes. During the fourth stage reconstruction takes place, vesicular resting daughter nuclei being formed out of the two groups of daughter segments, while the cell-body divides into two daughter cells. (From "The Cell," by Dr. Oscar Hertwig, 1895.) The manner of forming the "globular organic matrix" that Dr. Williams claims may be formed by the nucleus is here shown to be the process of the formation of new cells by nuclear segmentation.

These cells are formed to supply those that are necessary to cover the larger circumference of the enamel as it forms. I had formerly supposed, with many others, that new cells were supplied from the cells of the stratum intermedium. There are appearances which indicate this. But my recent investigation proves to my mind that

Fig. 7.

Section of developing tooth of pig, showing a layer of enamel substance folded over upon itself. The enamel cells have been teased away to show the fold.—*a*, fold of enamel substance; *b*, enamel cells, ameloblasts; *c*, developing tooth; *d*, developing bone of jaw.—Tolles, half-inch objective.

new cells needed in the ameloblastic layer originate wholly within the nucleus of the ameloblast. The new hypothesis, that the "globular organic matrix" which is to form the enamel rod has its origin from the nucleus, is probably altogether impossible. This statement is cautiously made. I have given the matter considerable attention, and have consulted several prominent biologists.

This globular, calcific matter is formed material. That which is formed by the nucleus of these specialized cells are *living daughter cells.* The power of the protoplasmic body to create different structures from the lime that it absorbs has more to do with the formation of these masses than the nucleus. It takes possession of

Fig. 8.

Section from developing tooth (human), showing the fibrous structure of the so-called "inner ameloblastic membrane."—*a*, a fibrous structure in which the enamel globules are deposited; *b*, *c*, enamel fibrils which form the substructure or scaffolding; *d*, formed enamel. —Hartnack, No. 9 objective, No. 2 eye-piece.

the lime-salts, forms them into calcific globules, and deposits them in the case of enamel calcification upon the outer surface of its calcifying structure.

In describing the probable origin of the enamel globule the following statement is made by Dr. Williams:

"It is not impossible that these bodies are of the same nature as the paranucleus or nebenkern of recent investigators in the field of cell mitosis." It is *not* probable that these investigators have studied the subject of cell mitosis from the specialized cells we call the ameloblasts. The term paranucleus or nebenkern is not used for the nucleus, but for a body, which is new, found in the develop-

Fig. 9.

Showing the first layer of enamel formed. The so-called "inner ameloblastic membrane." This is not a membrane. It is composed of a fibrous substructure in which the enamel globules are placed.—*a*, ameloblastic layer; *b*, substructure or scaffolding of fibres; *c*, dentine.—Hartnack, No. 9 objective, No. 2 eye-piece.

ment of spermatozoa, generally admitted to be formed from the remnants of spindle fibres after the last act of cell division preceding the formation of spermatozoon. It has also been used for other things. In this connection I offer a terse statement from a biologist of world-wide reputation, who writes, "In general I doubt the origin of many things which have been supposed to be budded off from the nucleus, and should want strong evidence to convince me that 'the globules' in question have thus originated."

We have thus briefly considered the source of the blood-supply, the so-called inner and outer ameloblastic membranes, the stratum intermedium, and the layer of ameloblasts of the enamel-forming

FIG. 10.

Section of forming enamel (human), showing the fibrous substructures.—*a*, connective tissue and stratum intermedium; *b*, the last formed layer of enamel, the so-called "inner ameloblastic membrane;" *c*, enamel fibrils, the substructure in which the globules are deposited; *d*, young enamel near dentine; *e*, dentine.—Hartnack, No. 9 objective, No. 2 eye-piece.

layers. I shall now ask your attention to the consideration of the structure of the youngest layer of enamel as it is formed before its full calcification. There is a period of growth here that has been but briefly considered. To me it is an important period, being that between the formation of the masses and of their becoming calcified into the enamel rods. It shows the transition period. It is doubtful if these stages of growth could be shown by the usual method of preparing sections. The bodies of calcific material that are deposited are found not always to be globular, seldom in the form of disks, more often oval or block-shaped, with the corners rounded, and are almost always larger than the enamel rod that is afterwards

FIG. 11.

Section of forming enamel (calf at birth), showing a fibrous substructure, or scaffolding, into which the globules are placed. The fibres are here shown as standing out like the teeth of a comb, cement substance being calcified about them.—Zeiss, 2 mm. objective, No. 12 eyepiece.

formed from this substance. They are deposited within what appears to be a semifluid, protoplasmic substance. As stated in my Atlanta paper, I think it is more than improbable that there are two sets of globules, each of a different nature. There are two distinct products, both of the same nature. They only differ morphologically. One is globular, the other fluid; each a somewhat different chemical combination not yet understood. If we wish to examine the so-called inner ameloblastic membrane, a transition tissue, and have been fortunate in making our sections as near the life of the tissue as possible, we shall see by using our highest powers precisely what I have formerly described. The masses at

Fig. 12.

Section of developing tooth (human), showing the structure of the so-called "inner ameloblastic membrane." At *a* are seen the globular bodies of uniform size and in orderly deposit; these are to form the enamel rods.—*a*, globular bodies forming rods; *b*, dentine.—Hartnack, No. 9 objective, No. 2 eye-piece.

the point of calcification are mostly oval or block-shaped, surrounded by a semisolid substance, which has been formed by the protoplasm of the ameloblast. This inter-rod cement is not formed by the globular masses, as recently stated. As the enamel globules are placed near the point of calcification, they are found to be somewhat larger than the rod they are to form. By some power they seem forced inward against the portion calcified, as if undergoing some sort of compression. Each mass is as yet separate from one another, with the semisolid cement substance around and between it. If this young enamel should now be teased away from the layer of ameloblasts, we should see where the epithelial fibres are. They stand out from the section somewhat like the teeth of a comb, but are so covered with the protoplasmic cement substance, which is partially calcified, that it masks them, and it is somewhat difficult to distinguish them as fibres; but they are too regular in their arrangement to be accidental formations, caused by the pulling out of calcific matter to form plasmic strings. If we examine another section, showing the enamel-forming layers, this transition layer, and the formed dentine, we shall find what appear to be fibres spanning any space from the ameloblasts to the formed dentine, in a regular, systematic order, as I shall show you this evening. The so-called plasmic strings, I am led to believe, are simply epithelial fibres. There is probably but little ground for the belief that they are formed by the protoplasm within the ameloblasts.

Let us now consider the substance that is called calcoglobulin, a name accepted by all investigators since the time of Rainey, Ord, and Harting. In his latest article, Dr. Williams, without any stated authority, says, "It has been pretty conclusively shown to be *not* calcoglobulin." He goes on to say, "Let not Dr. Andrews make the mistake of supposing that the experiments of Mr. Rainey, Dr. Ord, and Professor Harting have settled the problem of dentine and enamel formation. They are to be accepted for just what they are worth, and no more, and they fall a long way short of a complete explanation of the formation of these tissues in the living organism." This assertion is contrary to all the authorities of which I know. Professor Sudduth, whose opinion I value second to none, puts the matter in a different light when he says, "Mr. Rainey has by many and thoroughly scientific tests proved the analogy between his artificial calculi and those formed in the body. The lime-salts are deposited in both cases in a gelatin matrix. . . . The difference between crystallization outside of the body and crystallization within it is due to the action of the specially endowed cells. . . . On the border-land of calcification, between the completely fully calcified tissue and the formation matrix as yet unimpregnated with lime, there very constantly exists a stratum of tissue which in its physical and chemical properties very much resembles calcoglobulin." And again, "The secreted salts of calcium, which are thrown out by the cells, enter into chemical combination with the peripheral layer of protoplasm and form calcoglobulin, which, as we have before shown, is insoluble in acids."

Tomes says of it, "It belongs to that class of peculiar resistant substances which are to be found on the borders of calcification, and it behaves very much like Professor Harting's calcoglobulin." One can hardly expect us to accept Dr. Williams's unsubstantiated assertion until after it has been proved by careful and thoroughly scientific experiments. Does this authority wish to stake his reputation as an histologist on what he has said about calcoglobulin? I think not; his position cannot be an intentional one at all. It is probably the result of giving too little thought to a somewhat difficult subject. This appears on pages 126 and 127 of the *Dental Cosmos* for February, 1896, where, speaking of calcoglobulin, he says, "It is possible, and I think highly probable, that this substance, although appearing in the ameloblasts, is really formed in the more specifically secreting cells of the stratum intermedium." One who knew anything about the substance could hardly have made such a statement. Calcoglobulin is formed only at its point of calcification. See all authorities.

Another statement, on page 297 of the *Dental Cosmos* for April, 1896, is equally as carelessly made. It is where this statement occurs: "The larger, more transparent, and irregularly sized bodies of calcoglobulin [the term is not used "provisionally and with mental reservation" here] melt or flow together to form the interprismatic substance." Mark what follows. "This substance is more quickly destroyed by acid treatment than the enamel globules."

Authorities tell us that the line of calcoglobulin is held in some sort of chemical combination, for the last traces are retained very obstinately in this tissue, and it becomes exceedingly resistant to the action of acids, caustic, alkalies, and boiling water. This statement would prove to most of us that the globules spoken of which were not destroyed by acid strongly resemble calcoglobulin, while the material which he wrongly named calcoglobulin was easily destroyed. In that portion of the ameloblast farthest away from the calcifying enamel, calcific matter is being elaborated by mingling with the protoplasmic fluids. It is in such fine subdivisions that it is not often visible, even with our highest powers. A little lower in the region of the nucleus we have an ocular demonstration of it in the form of minute globules of calcific matter. These have been named calco-spherites, and they appear to enlarge not by growth, but by a process of coalescing, becoming larger as they are conveyed by the living matter of the cell to the region of the calcifying enamel. Here they are found to have coalesced into their typal form, very nearly of uniform size. At these times they are not the substance we call calcoglobulin. At the point of calcification they undergo an unknown chemical change of a calcifying nature. They become then a transition tissue,—a tissue that is seen on the borderland of calcification. It is exceedingly resistant to the action of acid, and is known to most authorities as the substance called calcoglobulin.

I see little in the third paper, in the *Dental Cosmos* for June, 1896, on formed enamel that I cannot heartily endorse, except the hypothesis advanced that the substances in the form of globular and block-shaped calcific masses which form the rods may have their origin from the nucleus, or that the plasmic strings have their origin within the protoplasm of the ameloblast. The following paragraph from Dr. Williams's text is an excellent word-picture: "If one were to make an elaborate pattern in clear glass, and then embed this pattern in other glass of lower melting-point, the problem of differentiating the pattern in clear glass would closely resemble the problem of differentiating the structure of completely formed enamel, and this illustration represents fairly well the manner in which enamel is formed. A somewhat elaborate pattern of translucent material is formed by the enamel cells."

This translucent material spoken of represents the substance of the enamel globules, which, by forming one over the other, will become the rod,—that is, by successive deposits.

Continuing, he writes, "Simultaneously or alternately with the formation of this pattern, another translucent substance of a more liquid character is formed, which flows or melts together all about the pattern-work, and the two become calcified together."

This translucent substance spoken of has in previous papers been mistaken for calcoglobulin, which it certainly is not. It represents the protoplasmic exudate from the ameloblasts that flows around the globules which are forming the rods, and becomes calcified as the cement substance. With these modifications, I can agree to the above statement. I am led to believe that the appearance of minute globular bodies within the calcified columns of the formed enamel, as seen in the illustrations in th June *Dental Cosmos*, are a form of arrested development. In sections of what appear to be enamel of finer structure we do not see these appearances. This third paper is of much value, and the illustrations are very beautiful. They show no structure with which I have not been somewhat familiar for many years. The structure, as seen in Fig. 82, I have demonstrated in all my lantern exhibitions since 1889.

In conclusion, I would say I believe that the formation of the enamel is in a sense a secreting process, but I do not believe secreting papillæ have been found in the stratum intermedium in all cases. This remains to be proved by further investigations. I believe there are two distinct products of the enamel-forming layers; that one of these products, from which the enamel rods themselves are built, is formed in the ameloblast, but not by nuclear formation. In the formed enamel rod the globular bodies are nearly or quite melted into one another at their extremities.

As the globular bodies pass from the ameloblasts they are placed in a net-work of what appears to be epithelial fibres, which pass within, between, and across the globular masses. Around the scaffolding thus formed the protoplasmic exudate flows, thus supplying the cement substance. Calcification then takes place, and enamel is formed. I believe, with Tomes, that enamel contains very little, if any, organic matter when fully calcified, and with Dr. Williams, that the finest lenses will not reveal the slightest difference between enamel ground from a living tooth and that which has lain in the earth for centuries.

Views which I have expressed in this paper differ somewhat from those of Dr. Williams. That he has made a number of misinterpretations seems evident. It is true these points in question cannot be considered as settled; this is a progressive subject, and I should be the last to claim my interpretations as wholly correct. Still less would I wish to ridicule the views of those from whom I differ. Antagonistic interpretations are necessary to the life and development of all scientific questions, and the truths are sooner proved in proportion to the diversity of opinion expressed by the investigators. Many conclusions are one-sided, and continually need correction. If, as is natural, I have placed my own interpretations in the foreground, I can also fully appreciate the immense work Dr. Williams has accomplished. With better instruments and better methods future investigators may be able to clear up all of these disputed points.

## Figures and Tables

**Fig. 1. f1:**
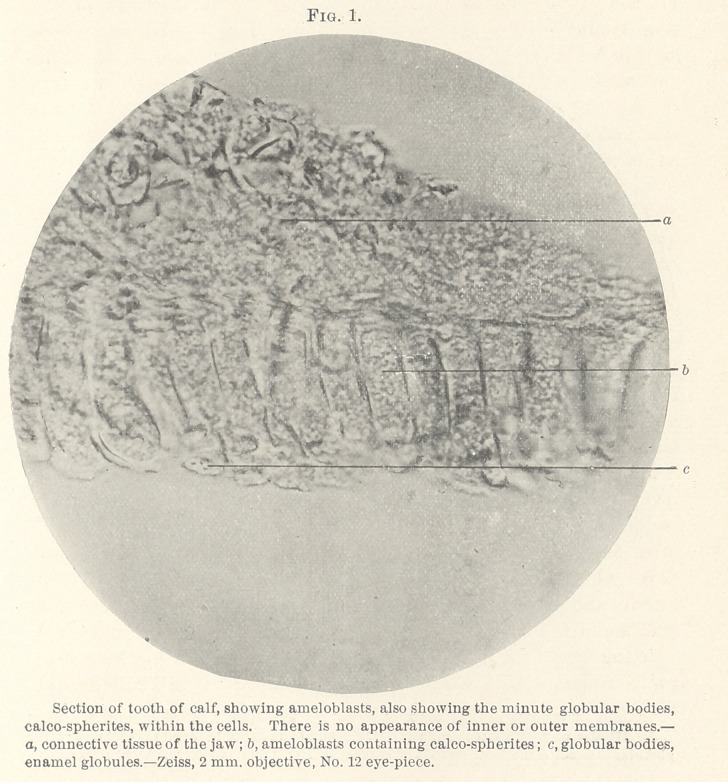


**Fig. 2. f2:**
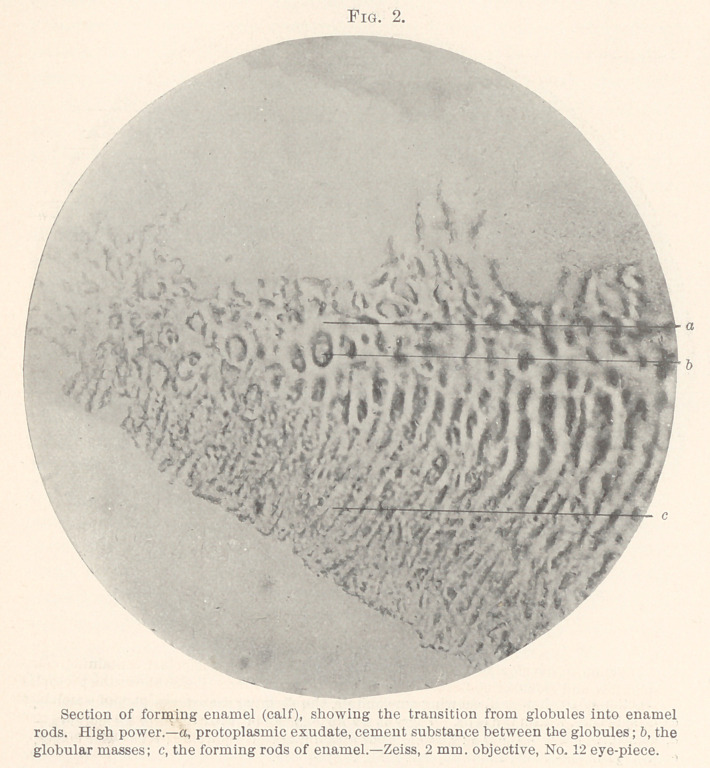


**Fig. 3. f3:**
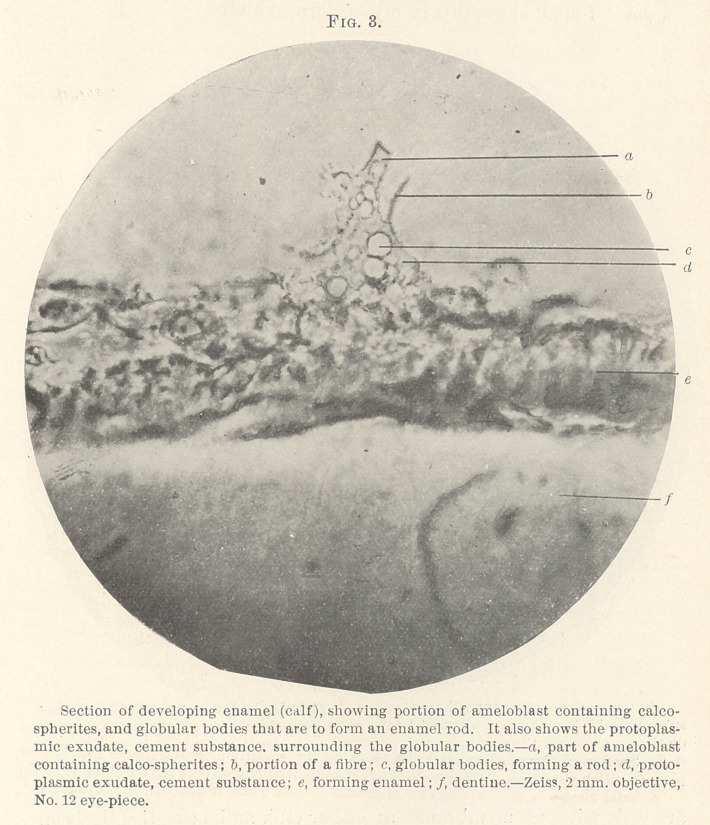


**Fig. 4. f4:**
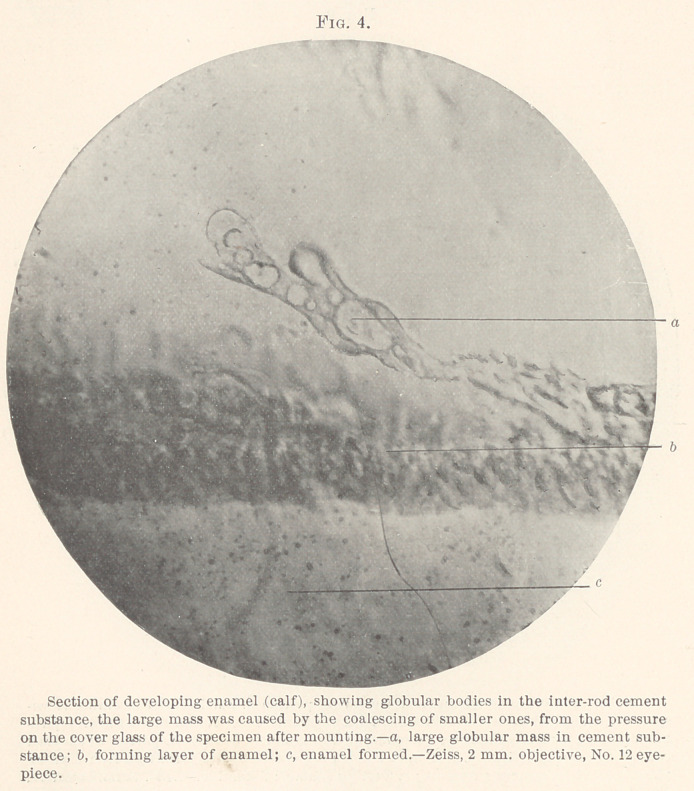


**Fig. 5. f5:**
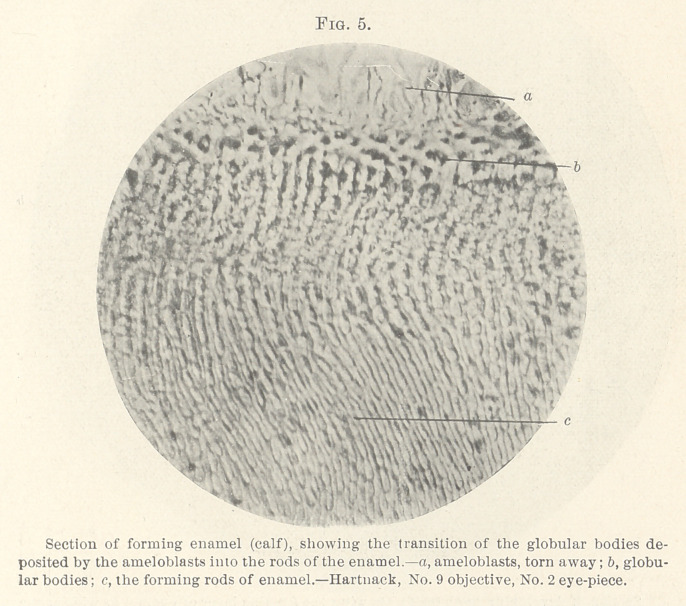


**Fig. 6. f6:**
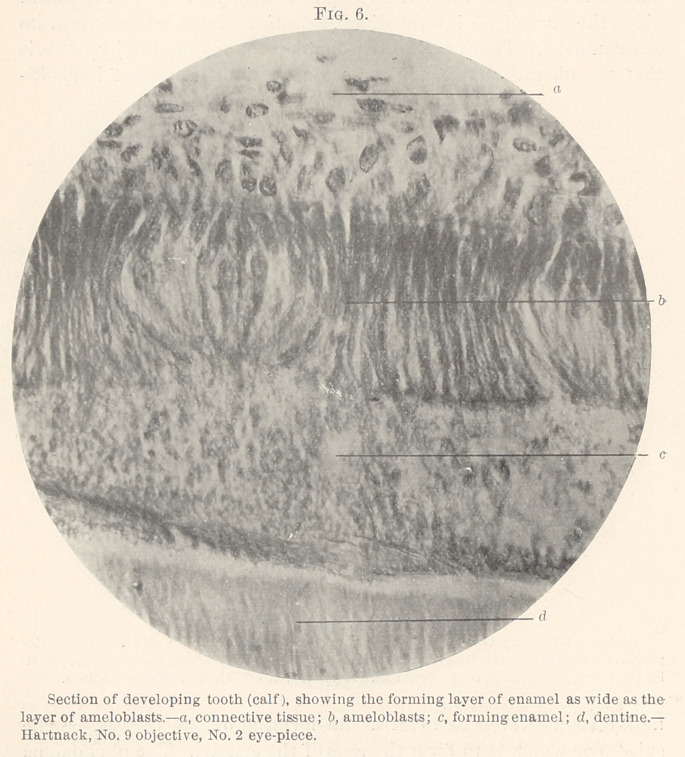


**Fig. 7. f7:**
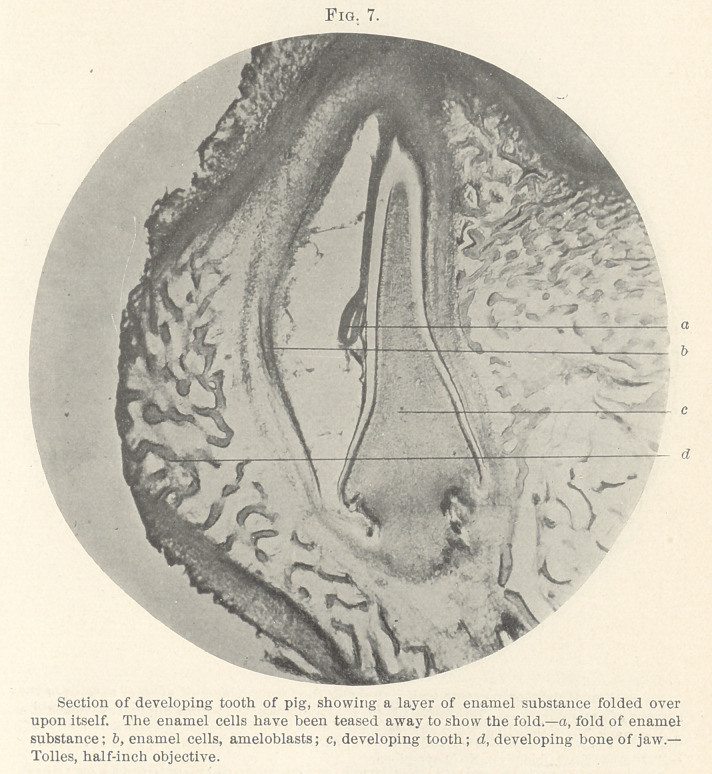


**Fig. 8. f8:**
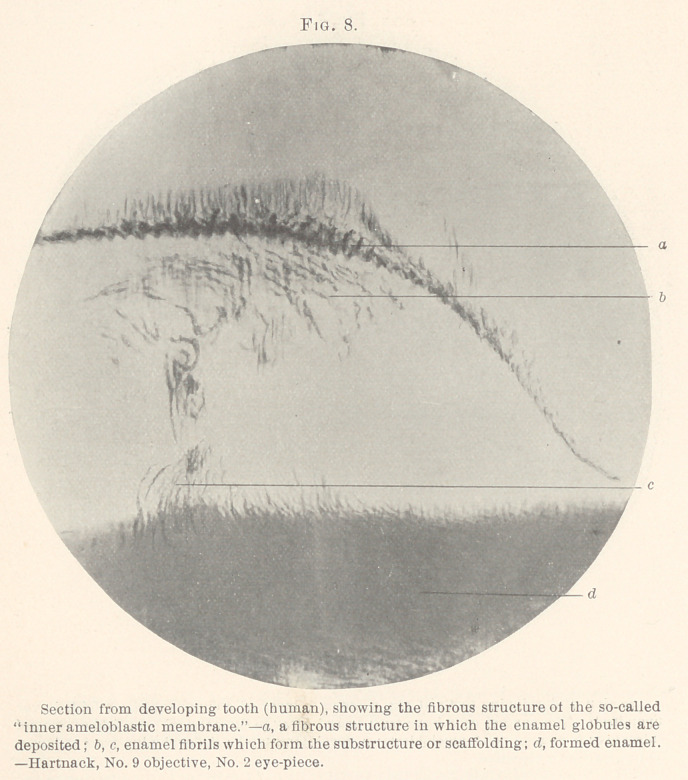


**Fig. 9. f9:**
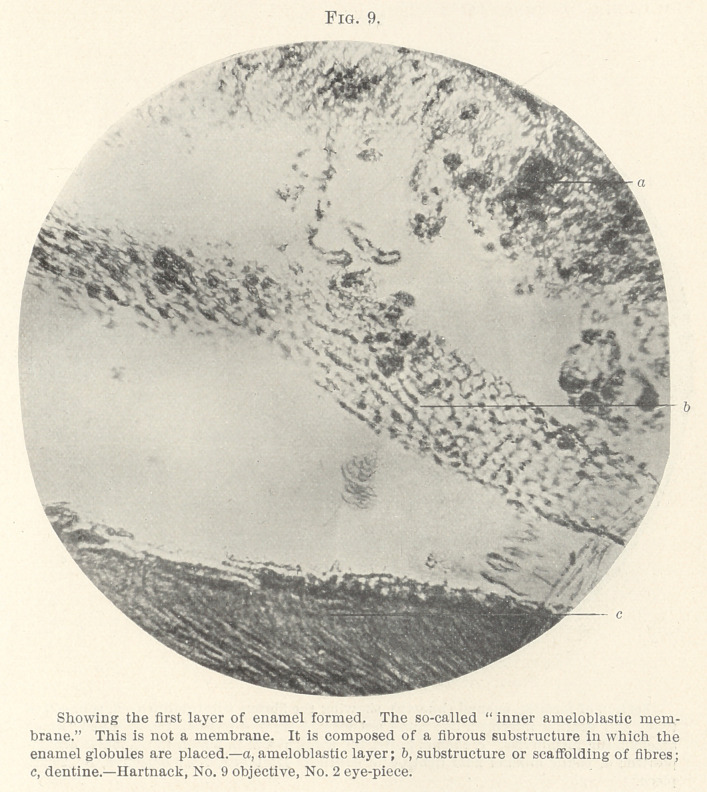


**Fig. 10. f10:**
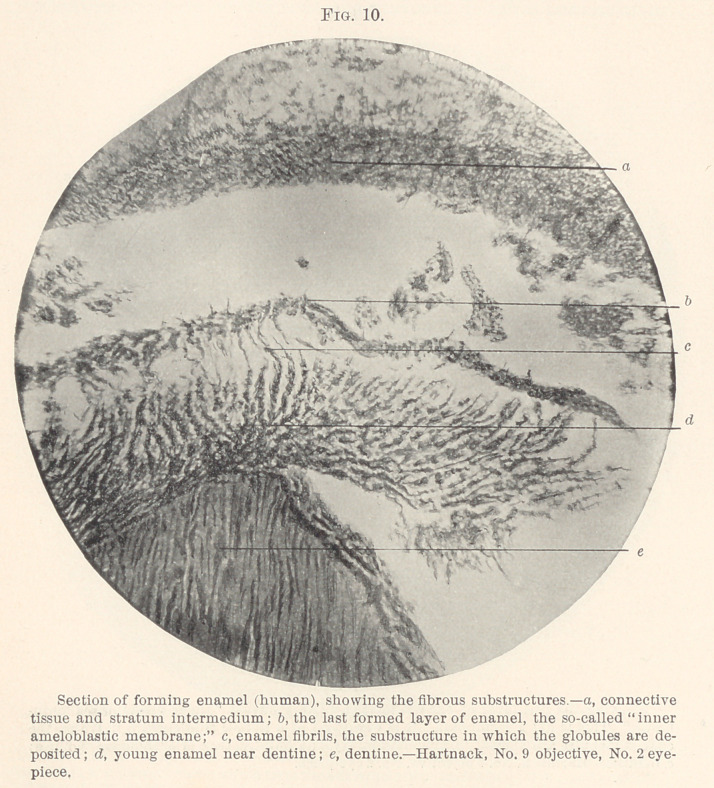


**Fig. 11. f11:**
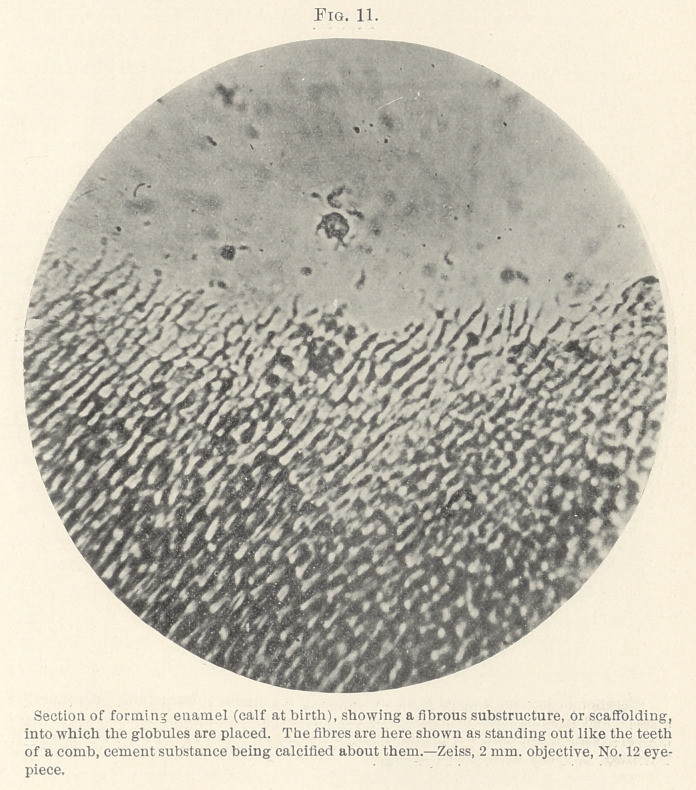


**Fig. 12. f12:**